# Phase I study of nanoliposomal irinotecan (PEP02) in advanced solid tumor patients

**DOI:** 10.1007/s00280-014-2671-x

**Published:** 2015-01-11

**Authors:** T. C. Chang, H. S. Shiah, C. H. Yang, K. H. Yeh, A. L. Cheng, B. N. Shen, Y. W. Wang, C. G. Yeh, N. J. Chiang, J. Y. Chang, L. T. Chen

**Affiliations:** 1 Department of Gynecology, Linkuo Chang-Gung Memorial Hospital, No.5, Fu-Hsing Street, Kuei-shan Hsiang, Taoyuan, 33305 Taiwan; 2 Cancer Center, Taipei Medical University Hospital, No.252, Wu Hsing Street, Taipei, 110 Taiwan; 3 Department of Oncology, National Taiwan University Hospital, No.1, Changde Street, Zhongzheng District, Taipei, 10048 Taiwan; 4 Department of Internal Medicine, National Taiwan University Hospital, No.1, Changde Street, Zhongzheng District, Taipei, 10048 Taiwan; 5 PharmaEngine, Inc., 16F, 237 Sung-Chiang Road, Taipei, 104 Taiwan; 6 National Institute of Cancer Research, National Health Research Institutes, No. 367, Sheng Li Road, Tainan, 70456 Taiwan; 7 Division of Hematology/Oncology, Department of Internal Medicine, National Cheng Kung University Hospital, College of Medicine, National Cheng Kung University, No.138, Shengli Road, Tainan, 70456 Taiwan; 8 Department of Internal Medicine and Cancer Center, Kaohsiung Medical University Hospital, Kaohsiung Medical University, No.100, Tzyou 1st Road, Kaohsiung, 807 Taiwan

**Keywords:** Irinotecan sucrosofate, Liposome, PEP02, MM-398, Pharmacokinetics, *UGT1A1* gene

## Abstract

**Purpose:**

To define the dose-limiting toxicity (DLT), maximum tolerated dose (MTD) and pharmacokinetics (PK) of PEP02, a novel liposome-encapsulated irinotecan, in patients with advanced refractory solid tumors.

**Methods:**

Patients were enrolled in cohorts of one to three to receive escalating dose of PEP02 in a phase I trial. PEP02, from 60 to 180 mg/m^2^, was given as a 90-min intravenous infusion, every 3 weeks.

**Results:**

A total of 11 patients were enrolled into three dose levels: 60 (one patient), 120 (six patients) and 180 mg/m^2^ (four patients). DLT was observed in three patients, one at 120 mg/m^2^ (grade 3 catheter-related infection) and two at 180 mg/m^2^ (grade 4 neutropenia lasting for >3 days in one, grade 4 hematological toxicities and grade 4 diarrhea in the other). MTD was determined as 120 mg/m^2^. Comparing with those after free-form irinotecan in the literature, the dose-normalized PK of SN-38 (the active metabolite) after PEP02 was characterized by lower *C*_max_, prolonged terminal half-life and higher AUC but with significant inter-individual variation. One patient who died of treatment-related toxicity had significantly higher *C*_max_ and AUC levels of SN-38 than those of the other three patients at 180 mg/m^2^. Post hoc pharmacogenetic study showed that the patient had a combined heterozygosity genotype of UGT1A1*6/*28. Two patients had objective tumor response.

**Conclusions:**

PEP02 apparently modified the PK parameters of irinotecan and SN-38 by liposome encapsulation. The MTD of PEP02 monotherapy at 3-week interval is 120 mg/m^2^, which will be the recommended dose for future studies.

## Introduction

It has been shown that topoisomerase-I (Topo I) is over-expressed in several cancer types, including breast, lung and colorectal cancers [1]. Irinotecan (CPT-11) is a water-soluble semisynthetic inhibitor of Topo I derived from camptothecin—a plant (*Camptotheca acuminata*) alkaloid, which can be converted to a more potent metabolite, SN-38, by carboxylesterase primarily in the liver. SN-38 can also be inactivated through glucuronidation by UDP-glucuronosyl transferase 1A1 (*UGT1A1*) to form SN-38G, which is mainly eliminated via biliary excretion. Both CPT-11 and SN-38 can bind to Topo I-DNA complex to interfere with the re-ligation of Topo I-induced single-strand DNA breaks and produce double-strand DNA damage during DNA synthesis [2]. Of them, SN-38 is approximately 100 to 1,000 times more potent than the CPT-11 as a Topo I inhibitor. Unfortunately, the metabolic conversions contribute to notable heterogeneities in both toxicity and efficacy of CPT-11, which lead to a rather narrow therapeutic index.

A liposome is a bilayer membrane spherical drug carrier vesicle that enables slow release of encapsulated drug so as to (1) lower drug elimination to prolong systemic circulation time, (2) lower maximum plasma concentration (*C*_max_) to reduce drug-associated side effects and (3) preferentially pass through the relatively large vascular pore openings in tumors to enhance its local accumulation in tumor tissue [3]. It has been known that both CPT-11 and SN-38 exist in a pH-dependent equilibrium between an inactive carboxylate form and an active lactone form after intravenous injection, and an acidic pH circumstance, for example in tumor microenvironment, will promote the formation of the active lactone form. Therefore, a liposome-encapsulated formulation will theoretically be able to shift the equilibrium toward more active lactone form formation within tumor tissue to enhance the treatment efficacy of CPT-11.

PEP02 is a novel nanoparticle formulation of irinotecan sucrosofate encapsulated with polyethylene glycolated liposome. The coupling of high molecular weight polyethylene-glycol (PEG) on the surface of PEP02 can effectively protect it from circulating protein binding and subsequent phagocytosis of the reticuloendothelial system to further enhance its circulation time. In preclinical animal studies, PEP02 showed improved preclinical pharmacokinetic properties and anti-tumor activity (in house data) [4]. Herein, we report the results of the first-in-human, phase I trial for PEP02 in patients with refractory advanced solid tumors. The objectives were to identify the maximum tolerated dose (MTD), dose-limiting toxicities (DLT) and safety profile, and to characterize the variables of pharmacokinetics (PK) of PEP02 administered as 90-min infusion every 3 weeks.

## Methods

### Trial design and patients

This trial was a multi-center, first-in-human, open-label, phase I, dose-escalation study of PEP02 (liposome-encapsulated irinotecan, PharmaEngine, Inc., Taipei, Taiwan), in patients with advanced refractory solid tumors. Patients with histologically confirmed advanced solid tumors that were refractory to standard systemic chemotherapy were eligible. Further inclusion criteria were age ≥20 years, Eastern Cooperative Oncology Group (ECOG) performance score of 0 or 1, life expectancy of more than 12 weeks and adequate bone marrow, hepatic and renal functions within 1 week before commencing treatment (hemoglobin ≥ 10 g/dL, absolute neutrophil count ≥1.5 × 10^3^/mL, platelets ≥ 100 × 10^3^/mL, serum bilirubin within normal limit, ALT ≤ 2.5× upper limit of normal, creatinine within normal limit). All prior active treatments, including major surgery, chemotherapy, radiotherapy (except palliative) or endocrine therapy, had to be ceased at least 4 weeks, and all treatment-related toxicities had to be resolved to no greater than grade 1 before enrollment. Patients with central nervous system metastases, pregnancy, uncontrolled active infection or other concomitant serious diseases and who had previously received irinotecan were excluded. All patients gave written informed consent. The trial was approved by the independent ethics committee of each participating institute and the Department of Health, Executive Yuan, Taiwan, and performed in accordance with International Conference on Harmonization Good Clinical Practice guidelines, Good Clinical Laboratory Practice and the Declaration of Helsinki.

### Treatment and assessment

PEP02 was diluted in 500 ml of 5 % dextrose and delivered as a 90-min intravenous infusion, every 21 days. Infusion time was allowed to be prolonged for acute infusion-associated reactions or any other clinical needs. Pre-medication included dexamethasone and serotonin-antagonist. Prophylactic anticholinergic agent was not given unless acute cholinergic reaction was observed in prior cycle of treatment. Anti-diarrhea agents were given according to the guideline of American Society of Clinical Oncology. After the infusion of PEP02, vital signs including blood pressure, pulse rate, respiratory rate and body temperature were monitored every 15 min for 3 h. Detailed history evaluation, vital signs recording, physical examination, complete blood count with differential classification and blood biochemistry tests were performed before treatment and weekly throughout treatment. Toxicity was recorded according to the National Cancer Institute-Common Toxicity Criteria (NCI-CTC) version 3.0, and a DLT was defined as any of the following events: grade 4 hematological toxicity lasting for longer than 3 days; febrile neutropenia; or >grade 3 non-hematological toxicity (except nausea and vomiting).

The starting dose of PEP02 was 60 mg/m^2^ (level I) based on the 1/10 of the LD10 in mice (the dose lethal to 10 % tested mice) and then would be escalated by 100 % (120 mg/m^2^, level II), 50 % (180 mg/m2, level III), 33 % (240 mg/m^2^, level IV), 25 % (300 mg/m^2^, level V), 16.7 % (350 mg/m^2^, level VI), and 11.4 % (400 mg/m^2^, level VIII), subsequently. The study was in a modified patient cohort accelerated titration design, in which single-patient cohorts for dose levels I–II, two-patient cohorts for levels III–IV and three-patient cohorts for level V or above would be recruited until any DLT was observed in the first cycle [5]. If a patient experienced any DLT, then additional patients would be recruited into that cohort. Dose escalation would be stopped if two or more of the patients experienced any DLT, and the prior dose level would be considered as the MTD. A minimum of six patients were required to be tested at the dose level defined as the MTD.

For patients who experienced grade 4 neutropenia and/or ≥grade 3 non-hematological toxicity, the dose of PEP02 would be reduced by one dose level in their subsequent cycle of treatment. Patients would receive PEP02 for a maximum of six courses, or until the presence of disease progression, unacceptable toxicity, treatment delay for ≥2 weeks, or patient’s refusal or death. Patients with tumor response or stable disease after six cycles of treatment could receive further PEP02 therapy in a compassionate use expansion program.

Imaging studies consisting of computed tomography of the abdomen and/or chest were performed before and after every two courses of chemotherapy to evaluate tumor response, which was determined according to the RECIST version 1.0 guidelines [6]. All complete and partial responses required confirmation by two consecutive observations no <4 weeks apart. Patients with a rapid objective or symptomatic progression before the next course of treatment were considered to have progressive disease (PD).

### Pharmacogenetic sampling and analyzing

Pharmacokinetic testing was done during the first course of PEP02 administration. Blood samples were collected before treatment, during the infusion at 30 and 60 min, at the end of infusion, and after infusion at 1, 2, 3, 6, 9, 12, 24, 48, 72 and 168 h. Plasma levels of encapsulated irinotecan, total irinotecan and SN-38 were determined by validated LC/MS/MS analytical methods. Pharmacokinetic parameters of individual data sets were analyzed by a non-compartmental model using WinNonlin Professional version 4.1 (Pharsight Corporation, Menlo Park, CA).

Peak concentration in plasma (*C*_max_) and the time to achieve *C*_max_ (*T*_max_) were determined directly by a visual analysis of the individual observed plasma concentration versus time curve data. Area under the plasma concentration–time curve from time zero to infinity (AUC_0→∞_) was determined by the trapezoidal rule and extrapolated to infinity, which was estimated by the last quantifiable concentration divided by the terminal elimination rate constant *λ*_Z_ (*K*_el_). *λ* was determined by a simple log-linear regression based on the last three points of plasma concentration. Total clearance of drug from plasma (Cl) was determined by the dose divided by the AUC. Plasma terminal elimination half-life (*t*_1/2_) was calculated by dividing *λ* into the natural logarithm of two. Mean residence time from time zero to infinity (MRT_0→∞_) was calculated from the area under the first moment curve from time zero to infinity (AUMC_0→∞_) divided by AUC_0→∞_. Volume of distribution at steady state (*V*_ss_) was determined by MRT × Cl.

### Statistical analysis

The association between discrete variables was assessed using Fisher’s exact test. The two-tailed Wilcoxon rank sum test was used for the comparison of pharmacokinetic parameters. A value of *p* < 0.05 was considered statistically significant.

## Results

### Patient characteristics, dose escalation, DLT and MTD

Between January 2005 and August 2005, a total of 11 patients (median age 47, range 41–67 and ECOG PS of 0 or 1) were enrolled. The demographics and baseline characteristics of all patients are listed in Table 1. These patients were enrolled into three dose levels, with 1, 6 and 4 patients in dose level I, II and III, respectively (Table 2). Initially, none of the first two patients who were separately enrolled at dose level I and level II experienced a DLT. In dose level III (180 mg/m^2^), because one of the three patients (Patient #103) developed a DLT (grade 4 neutropenia lasting for longer than 3 days), the study cohort was expanded. The first additionally enrolled patient (Patient #203) also had DLTs (grade 4 febrile neutropenia, grade 4 thrombocytopenia with bleeding event and grade 4 diarrhea) so that dose escalation was stopped and five more patients were enrolled at dose level II (120 mg/m^2^). Among the total of 6 patients at dose level II, only one patient (Patient #205) experienced a DLT (grade 3 catheter-related infection); thus, 120 mg/m^2^ was determined to be the MTD.

**Table 1:** Patient characteristics

Characteristic	Patients, *n* (%)
Patients enrolled	11
Age (years)	
Median	47
Range	41–67
Sex	
Male	1 (9)
Female	10 (91)
ECOG performance status	
0	6 (55)
1	5 (45)
Tumor type	
Cervical cancer	4 (36)
Breast cancer	2 (18)
Neuroendocrine tumor	2 (18)
Pancreatic cancer	1 (9)
Non-small cell lung cancer	1 (9)
Thymic carcinoma	1 (9)
Previous treatment	
Surgery	9 (82)
Radiotherapy	8 (73)
Chemotherapy	11 (100)

**Table 2:** Dose-escalation schedule and enrolled patient number

Dose level	Dose (mg/m^2^)	Proposed patient numbers	Actual patient numbers
I	60	1	1
II	120	1	6
III	180	2	4
IV	240	2	0
V	300	3	0
VI	350	3	0
VII	400	3	0

### Drug delivery and adverse events

A total of 40 courses of chemotherapy were delivered, with a median of four courses per patient (range, 1–6 courses). Treatment delay and dose modification were required in 5 (45.5 %) and 2 (18.2 %) patients, respectively. Of the latter two patients, one had dose reduction from 180 to 120 mg/m^2^ and to 60 mg/m^2^ because of hematological toxicities, and the other from 120 to 60 mg/m^2^ due to grade 3 nausea and vomiting. Table 3 shows the drug-related adverse events experienced in at least two patients. The most common toxicity observed in the six patients at the MTD dose level (120 mg/m^2^) was diarrhea (100 % in all grades, 33 % in grade 3/4) and vomiting (83.3 % in all grades, 66.7 % in grade 3/4). There was no anaphylactic allergic or severe infusion reaction occurred in this phase I study. Only one (#301) patient experienced chest tightness after 30 min of infusion during cycle 2 treatment, but corresponding vital signs were stable. No other infusion reactions were reported on the following cycle.

**Table 3:** Drug-related adverse events

	60 mg/m^2^ *N* = 1		120 mg/m^2^ *N* = 6		180 mg/m^2^ *N* = 4	
	All grade *N* (%)	G 3/4 *N* (%)	All grade *N* (%)	G 3/4 *N* (%)	All grade *N* (%)	G 3/4 *N* (%)
Diarrhea	1 (100)	0 (0)	6 (100)	2 (33.3)	4 (100)	1 (25)
Vomiting	1 (100)	0 (0)	5 (83.3)	4 (66.7)	2 (50)	2 (50)
Nausea	1 (100)	0 (0)	4 (66.7)	2 (33.3)	2 (50)	1 (25)
Alopecia	0 (0)	NA	3 (50)	NA	3 (75)	NA
Fatigue	1 (100)	0 (0)	3 (50)	1 (16.7)	1 (25)	0 (0)
Leukopenia	0 (0)	0 (0)	2 (33.3)	1 (16.7)	2 (50)	2 (50)
Neutropenia	0 (0)	0 (0)	2 (33.3)	1 (16.7)	2 (50)	2 (50)
Weight decreased	1 (100)	0 (0)	2 (33.3)	0 (0)	1 (25)	0 (0)
Dizziness	0 (0)	0 (0)	2 (33.3)	0 (0)	0 (0)	0 (0)
Anemia	0 (0)	0 (0)	1 (16.7)	0 (0)	1 (25)	0 (0)
Anorexia	0 (0)	0 (0)	1 (16.7)	0 (0)	1 (25)	0 (0)
Electrolyte imbalance	0 (0)	0 (0)	1 (16.7)	0 (0)	1 (25)	0 (0)

There was one treatment-related death at dose level III (180 mg/m2). A 67-year-old female patient (Patient #203) with poorly differentiated neuroendocrine tumor (small cell carcinoma) of the pancreas developed severe watery diarrhea and neutropenic fever (WBC and ANC of 360 and 4/mm^3^, respectively) 8 days after her first dosing of PEP02. Despite empiric antibiotics, granulocyte-colony stimulating factor (G-CSF) and anti-diarrhea therapy, she died of septic shock, disseminated intravascular coagulopathy and acute respiratory distress syndrome 7 days later. The event was likely related to the alterations of PEP02 PK secondary to the presence of combined heterozygosity of irinotecan metabolism-related genetic polymorphisms of *UGT1A1* as described later.

### Pharmacokinetic and exploratory pharmacogenetic studies

**Fig. 1:**
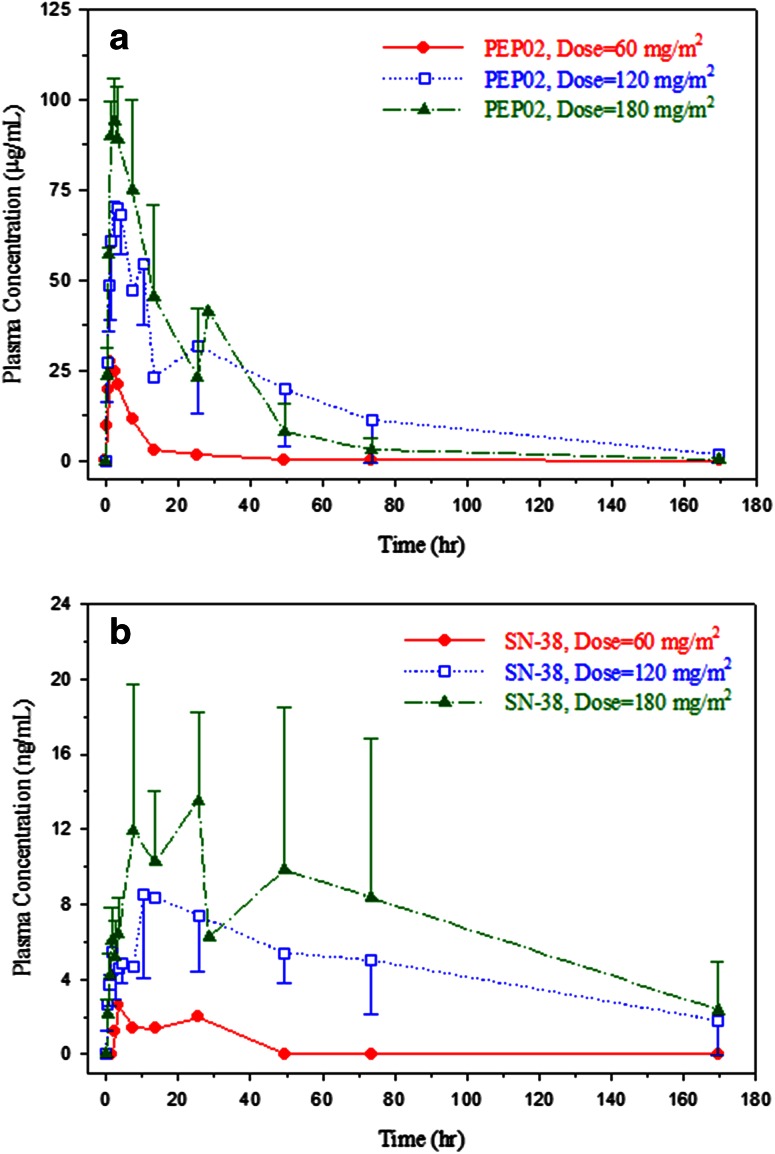
Plasma concentration–time profiles of **a** encapsulated CPT-11 (PEP02) and **b** SN-38 at 60, 120 and 180 mg/m^2^ dose level of PEP02

**Fig. 2:**
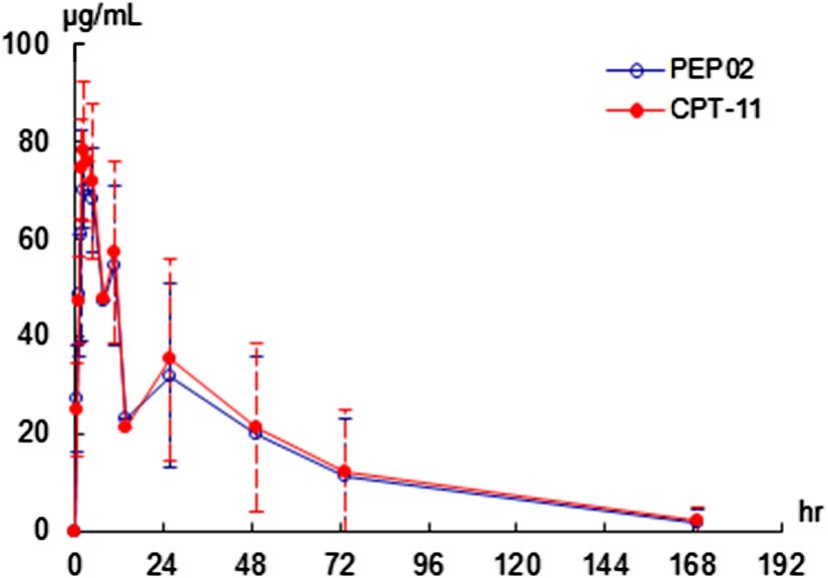
Plasma concentration–time profiles of encapsulated CPT-11 (PEP02) and total CPT-11 at 120 mg/m^2^ dose level of PEP02

The PK of PEP02 is listed in Table 4 and graphed in Fig. 1a, b. The PK parameters of CPT-11 PEP02 dosing, i.e., after 120 mg/m^2^, were characterized by slow clearance (mean = 0.0591 L/m^2^/h), small volume of distribution (mean = 1.8 L/m^2^ ≅ plasma volume) and prolonged terminal half-life (mean = 29.5 h). In addition, the plasma concentration–time profile of encapsulated irinotecan (PEP02) in each patient matched approximately with that of total irinotecan (Fig. 2). The results suggest that the release of irinotecan from liposomes occurred slowly over time. The *C*_max_, terminal *t*_1/2_ and AUC of SN-38 after 120 mg/m^2^ of PEP02 were 9.2 ± 3.5 ng/mL, 75.4 ± 43.8 h and 710 ± 395 ng*h/mL, respectively. However, the correlations between *C*_max_ or AUC_0–∞_ of SN-38 and PEP02 doses were weak (*r* ^2^ = 0.423 for *C*_max_ vs. PEP02 dose; *r* ^2^ = 0.0652 for AUC_0–∞_ vs. PEP02 dose). The elimination of SN-38 was much slower and presented larger inter-individual variability than those of PEP02 and irinotecan. With the small number of patients and interpatient variability, it is difficult to conclude the dose-proportionality PK of CPT-11 between the three dose levels. However, higher AUC was observed at higher dose levels.

**Table 4:** Pharmacokinetic parameters of PEP02 at each dose level

	Dose level (mg/m^2^)	*C*_max_ (*u*g /mL) CPT-11(ng /mL) SN-38	*T*_max_ (hr)	AUC_0–169.5_ (hr-*u*g/mL) CPT-11 (hr-ng/mL)SN-38	AUC_0–∞_ (hr-*u*g/mL) CPT-11 (hr-ng/mL)SN-38	*V*_ss_ (L/m^2^)	Cl (L/hr/m2)	*t*_1/2_ (hr)	MRT_0–∞_ (hr)
Total CPT-11	60, *N* = 1	31.8	1.5	222	223	3.56	0.269	28.7	13.2
	120, *N* = 6	79.4 ± 13.9	2.5 ± 1.1	2,835 ± 1,817	2,963 ± 1,947	1.8 ± 0.771	0.0591 ± 0.0367	29.5 ± 17.2	38.6 ± 19.5
	180, *N* = 4	102 ± 17.6	1.75 ± 0.5	1,945 ± 1,029	1,963 ± 1,035	1.97 ± 0.342	0.119 ± 0.0703	22.3 ± 11.5	20.5 ± 9.47
SN-38	60, *N* = 1	2.58	3.6	38.4	NC	NA	NA	NC	NC
	120, *N* = 6	9.20 ± 3.50	21.9 ± 26.3	710 ± 395	997 ± 680	NA	NA	75.4 ± 43.8	109.0 ± 54.4
	180, *N* = 4	14.3 ± 6.16	21.0 ± 9.0	1,159 ± 969	1,425 ± 1,134	NA	NA	58.0 ± 32.8	90.9 ± 43.1

Mean ± SD; *C*_*max*_ peak concentration in plasma; *T*_*max*_ time to achieve peak plasma concentration; *AUC*_*0–169.5*_, *AUC*_*0–∞*_ area under the plasma concentration–time curve from time zero to 169.5 h and infinity, respectively; *V*_*ss*_ volume of distribution at steady state; *t*_*1/2*_ plasma terminal elimination half-life; *Cl* total clearance of drug from plasma; *MRT*_*0–∞*_ mean residence time from time zero to infinity; *NC* not calculated because there was no distinct terminal log-linear phase for the *λ*_z_ determination; *NA* not available

**Fig. 3:**
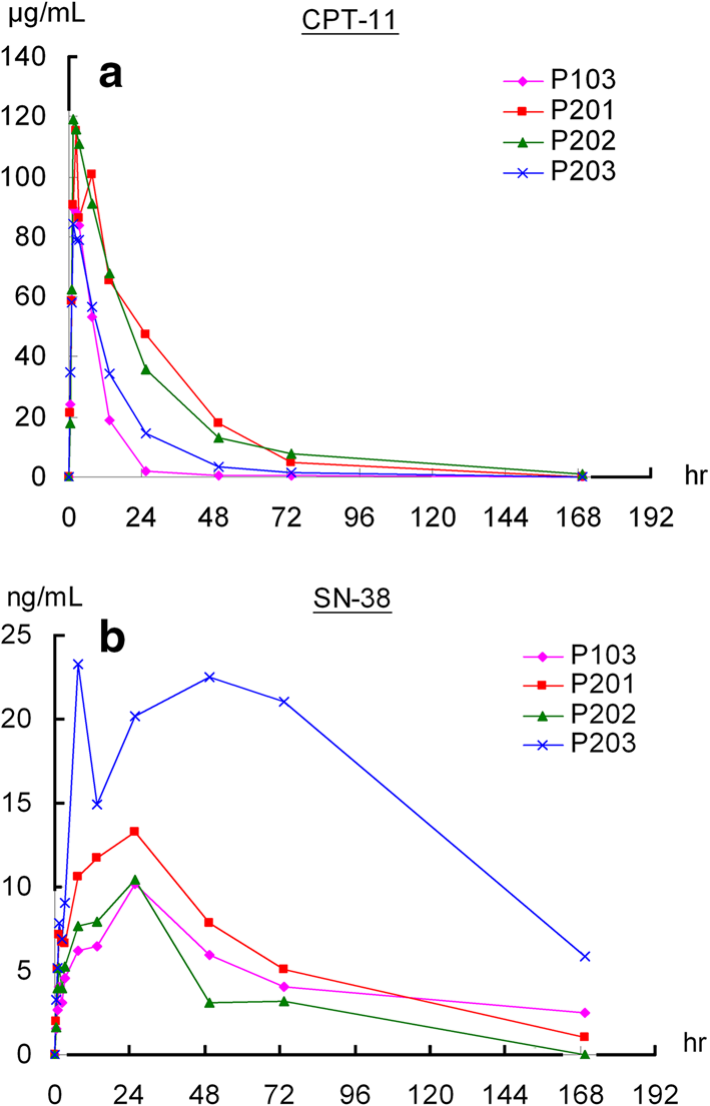
Plasma concentration–time profiles of **a** total CPT-11 and **b** SN-38 in subjects at 180 mg/m^2^ dose level of PEP02

The AUC_0–∞_ and *C*_max_ of CPT-11 and SN-38 of the patient who died of treatment-related complications (Patient #203) were investigated as shown in Fig. 3a, b. The AUC_0–∞_ and *C*_max_ of SN-38 of patient #203 were 2–3 folds higher than those of the other three patients receiving the same dose of treatment. To explore the potential genetic background for the differences, the irinotecan metabolism-related genetic polymorphisms of patients receiving 180 mg/m^2^ were determined after the approval of IRB. Three of the four patients with available stocked peripheral blood mononuclear cells were included in the pharmacogenetic study, which showed that the patient (Patient #203) who died of treatment-related toxicity had combined heterozygosity of UGT1A1*6/*28 (Table 5).

**Table 5:** Pharmacokinetic parameters of SN-38 and pharmacogenetic data of patients received 180 mg/m^2^ of PEP02

Patient unique number	AUC_0–∞_ (hr-ng/mL)	*C*_max_ (ng /mL)	*t*_1/2_ (hr)	UGT1A1*6	UGT1A1*28
201	906	13.3	41.4	W/W	W/W
202	549	10.4	28.2	W/W	W/W
203	3,084	23.3	59.0	V/W	V/W
103	1,159	10.2	104	ND	ND

*V* variant; *W* wild type

*ND* not done

### Efficacy

The best tumor response was partial response in two out of 11 intent-to-treat (ITT) patients (18.2 %) or out of ten response evaluable patients (20 %). One patient with pancreatic cancer who failed to several lines of treatment including gemcitabine and infusional 5FU/LV alone or in combination with oxaliplatin had PEP02 at the dose of 180 mg/m^2^, and the other one with cervical cancer whose tumor relapsed after cisplatin-based concurrent chemo-radiotherapy had PEP02 at the dose of 120 mg/m^2^. Another three patients with breast cancer, pancreatic neuroendocrine tumor and thymic carcinoma in each had stable disease. Therefore, the disease control rate was 45.5 % for ITT population or 50 % for response evaluable patients. At the MTD dose of 120 mg/m^2^, 5 out of 6 patients were evaluable for tumor response. One patient (#205) with squamous cell carcinoma of the lung who was early off-studied because of prolonged treatment interruption secondary to DLT (grade 3 catheter-related infection) was excluded from response evaluation. The response rate and disease control rate of evaluable patients were 20 and 60 %, respectively. However, the unevaluable patients (#205) received five additional courses of PEP02 at 120 mg/m^2^ after adequate infection control and achieved a partial response under the compassionate use program.

## Discussion

The current study has established the MTD, safety profile, PK and preliminary efficacy of PEP02, a nanoliposomal formulation of irinotecan, in patients with refractory advanced cancer. Myelosuppression and diarrhea were the major DLTs, and 120 mg/m^2^ was defined as the MTD. The toxicity pattern seems to be comparable with that of free-form irinotecan [7, 8]. Of note, in the absence of prophylactic atropine administration, there was only one episode of grade 1 acute cholinergic syndrome (abdominal pain) observed in this study, as compared to the occurrence in 9 of 23 patients who received free irinotecan ≥240 mg/m^2^ in a phase I trial [9]. It has been shown that irinotecan may inhibit acetyl-cholinesterase to enhance parasympathetic discharge, and the frequency and severity of cholinergic syndrome are likely irinotecan concentration dependent [10, 11].

In the current study, pharmacokinetic analysis demonstrated that the plasma concentration–time profile of PEP02 (encapsulated irinotecan) in each patient matched approximately with that of total irinotecan, indicating that the release of free-form irinotecan from the nanoliposomes occurred slowly over time. We were not able to measure plasma level of free CPT-11 directly because it was below the lower detection limit of the LC/MS/MS assay. The slow release of irinotecan from PEP02 resulted in small volume of distribution (mean = 1.8 L/m^2^ ≅ plasma volume), slow clearance and prolonged terminal half-life of circulating total irinotecan, and a favorable PK of its active metabolite, SN-38. Comparing the PK of SN-38 in this study with the published studies following administration of 125 mg/m^2^ free-form irinotecan in the literature, the PK parameters of SN-38 after 120 mg/m^2^ of PEP02 showed lower *C*_max_ (9.2 ± 3.5 vs 26.3 ± 11.9 ng/mL), longer terminal *t*_1/2_ (75.4 ± 43.8 vs 10.4 ± 3.1 h) and higher AUC (710 ± 395 vs 229 ± 108 ng*h/mL) [12, 13]. The AUC of SN-38 after 120 mg/m^2^ PEP02 was roughly comparable with that achievable with 300–350 mg/m^2^ of “conventional” irinotecan in the literature. The lower toxicity profile potentially makes PEP02 a better agent to combine with other cytotoxic agents, i.e., 5-fluorouracil and folinic acid, and/or targeted agents, i.e., bevacizumab or cetuximab for advanced colorectal cancer. However, the optimal dosages of PEP02 for such combinations remain to be determined.

The correlations between the *C*_max_ or AUC_0–∞_ of SN-38 and doses of PEP02 were weak in this phase I study. The elimination of SN-38 was slow and exhibited significant inter-individual variation after administration of PEP02. The reason for such inter-individual variation in kinetic behavior of SN-38 after PEP02 administration is not yet fully explored, but pharmacogenetic variability of irinotecan metabolism-related enzymes is likely to be involved. The presence of the UGT1A1*28 allele has been shown to cause a 70 % reduction in the expression of UGT, the enzyme responsible for glucuronidation of SN-38 into inactive SN-38 glucuronide (SN-38G). This reduction leads to increased exposure of patients to the cytotoxic metabolite, SN-38 [14, 15]. Clinically, patients with either heterozygous UGT1A1*1/*28 or homozygous UGT1A1*28/*28 genotypes are more prone to severe irinotecan-associated toxicity, notably grade 3–4 neutropenia and/or diarrhea [15]. Based on these findings, the US Food and Drug Administration revised the label of irinotecan and recommended that patients who are known to be homozygous for the UGT1A1*28 allele should receive a reduced initial dose of irinotecan to minimize the risk of significant toxicity [16]. However, ethnic differences in *UGT1A1* allele frequencies are well established, and the Asian population is known to have a lower frequency of the UGT1A1*28 allele (13.9 % vs 33.4 % in Caucasians) but a significantly higher frequency of UGT1A1*6 (13.0 % vs 0.5 % in Caucasians) [15]. It has been reported that patients with a combined heterozygosity of UGT1A1*6 and *28 were more prone to develop toxicities after irinotecan injection, as happened in one of our patients [17, 18]. Comparing the PK of SN-38 in the four patients receiving 180 mg/m^2^ of PEP02, the *C*_max_ and AUC_0–∞_ of SN-38 of the patient (#203) who died of grade 4 diarrhea, neutropenia and infection were almost threefold higher than in the other three patients.

Other liposome formulations of irinotecan or SN-38 have also been developed. IHL-305 (pegylated liposomal irinotecan) has been identified in its MTD at every 4 week and every 2 week schedule as 160 and 80 mg/m^2^, respectively, in a phase I study [19]. The AUC_0–∞_ of SN-38 at 160 mg/m^2^ was 360 ± 370 ng*h/mL, and one PR and two SD were observed among the 60 patients recruited. PEP02 at 120 mg/m^2^ showed higher SN-38 exposure than IHL-305 at 160 mg/m^2^. LE-SN38 is a liposome-encapsulated SN-38, which had been developed in phase II stage. The MTD of LE-SN38 was identified in its phase I study as 35 mg/m^2^ every 3 weeks for both the UGT1A1*28 wild-type and heterozygous patients [20]. The AUC_0–∞_ of SN-38 at the MTD for the wild-type and heterozygous patients were 1,751.8 and 3,493.6 ng*h/mL, respectively. Notwithstanding LE-SN38 has relatively high SN-38 AUC, unfortunately, it did not meet the pre-specified activity criteria in its phase II CALGB 80402 study for mCRC patients [21].

Although antitumor activity was not the primary endpoint in this phase I trial, two patients with partial response and three patients with stable disease were observed out of 11 ITT patients. Notably, one patient (#205) who developed non-drug-related toxicity after the first course and received five additional courses under compassionate use program also had partial response. Several researches investigating the efficacy of PEP02 with or without other anticancer drugs are currently ongoing. In conclusion, the MTD of PEP02 given every 3 weeks is 120 mg/m^2^, and major treatment-related DLTs are myelosuppression and diarrhea. Promising anti-tumor activities that were observed in the patients who were refractory to available treatments warrant further clinical investigations.
